# Single crystal elasticity of natural topaz at high-temperatures

**DOI:** 10.1038/s41598-017-17856-3

**Published:** 2018-01-22

**Authors:** Sumudu Tennakoon, Ye Peng, Mainak Mookherjee, Sergio Speziale, Geeth Manthilake, Tiglet Besara, Luis Andreu, Fernando Rivera

**Affiliations:** 1 Earth Materials Laboratory, Department of Earth, Ocean and Atmospheric Sciences, Florida State University, Tallahassee, FL 32306 USA; 2 GFZ, German Research Centre for Geosciences, 14473 Potsdam, Germany; 3 Université Clermont Auvergne, CNRS, IRD, OPGC, Laboratoire Magmas et Volcans, F-63000 Clermont-Ferrand, France; 4 National High Magnetic Field Laboratory, Tallahassee, FL 32310 USA

## Abstract

Topaz is an aluminosilicate mineral phase stable in the hydrothermally altered pegmatitic rocks and also in subducted sedimentary lithologies. In nature, topaz often exhibits solid solution between fluorine and hydrous end members. We investigated elasticity of naturally occurring single crystal topaz (Al_2_SiO_4_F_1.42_(OH)_0.58_) using Resonant Ultrasound Spectroscopy. We also explored the temperature dependence of the full elastic constant tensor. We find that among the various minerals stable in the Al_2_O_3_-SiO_2_-H_2_O ternary system, topaz exhibits moderate elastic anisotropy. As a function of temperature, the sound velocity of topaz decreases with $\frac{{\boldsymbol{d}}{{\boldsymbol{V}}}_{{\boldsymbol{P}}}}{{\boldsymbol{d}}{\boldsymbol{T}}}$ and $\frac{{\boldsymbol{d}}{{\boldsymbol{V}}}_{{\boldsymbol{S}}}}{{\boldsymbol{d}}{\boldsymbol{T}}}$ being −3.10 and −2.30 × 10^−4^ km/s/K. The elasticity and sound velocity of topaz also vary as a function of OH and F content. The effect of composition (${{\boldsymbol{x}}}_{{\boldsymbol{O}}{\boldsymbol{H}}}$) on the velocity is equally important as that of the effect of temperature. We also note that the Debye temperature (${{\boldsymbol{\Theta }}}_{{\boldsymbol{D}}}$) of topaz at room temperature condition is 910 K and decreases at higher temperature. The Debye temperature shows positive correlation with density of the mineral phases in the Al_2_O_3_-SiO_2_-H_2_O ternary system.

## Introduction

Topaz is an aluminosilicate mineral with a general stoichiometry of Al_2_SiO_4_F_2*x*_(OH)_2(1*−x*)_, with *x* = 0 representing hydroxyl end-member of topaz and $x$ = 1 representing fluorine end-member of topaz. Topaz is also one of the principal fluorine bearing silicates that occur in fluorine rich pegmatitic rock^1–4^. Hydroxyl components in topaz are enriched in ultrahigh-pressure metamorphic rocks^2,5^. In addition, experimental studies on the Al_2_O_3_-SiO_2_-H_2_O ternary system that represents subducted hydrated sediments revealed end-member topaz-OH (Al_2_SiO_4_(OH)_2_) as a likely phase stable up to a pressure of around ~12 GPa^6–9^. Thus, the hydroxyl rich topaz might play a significant role in transporting water in to the deep Earth^9^.

In order to relate the degree of mantle hydration caused by the subduction of hydrated sedimentary rocks, we need a better constraint on the elastic properties of the constituent mineral phase. Thus, elasticity of fluorine bearing topaz has been examined at ambient conditions^10^. More recently pressure dependence of the full elastic constant tensor of topaz-OH has been explored using *first principles* method based on density functional theory^11^. Effect of OH = F substitution on the elasticity has also been explored using *first principles* simulation^12^. However, the effects of temperature on the elasticity of topaz remains poorly explored. In a recent study, the temperature dependence of a natural topaz has been explored up to 312 K^13^. In this study, we explore the effect of temperature on the full elastic tensor of a natural topaz up to 973 K, temperature relevant for the Earth’s interior.

## Method

We determined the lattice parameters, *a* = 4.66217(12) Å, *b* = 8.82299(19) Å, *c* = 8.3869(2) Å, for a natural crystal of topaz using single crystal x-ray diffraction (Supplementary Figure SF1). We used an Oxford-Diffraction Xcalibur-2 CCD diffractometer with molybdenum (Mo) Kα radiation (λ = 0.71 Å). We used the X-ray diffraction facility at the National Magnetic High Field Laboratory, Florida State University. Topaz has orthorhombic space group symmetry *Pbnm*. We used the single crystal X-ray diffraction to orient the [001], [010], and [001] with crystal axes parallel to the axes of the rectangular prallepiped geometry. To determine the orientation of single-crystal, we mounted the crystal on a four-axis Enraf-Nonius CAD-4 Single Crystal X-ray Diffractometer. The orientation matrix for the single crystal is precise within ~$\pm 1^\circ$. We determine the mass of the single crystal of topaz in air ~0.0515 g and in water ~0.0369 g with a specific gravity of ~3.523. The density determined using a combination of methods is ${\rho }_{0}$ ~3.520(4) g cm^−3^.

We determined the chemical composition of topaz by electron probe micro analysis (EPMA) using a JEOL JXA-8200 electron microprobe operating at an accelerating voltage of 15 kV, beam current of 15 nA, and spot size of 20 μm. We determined the stoichiometry of the topaz to be Al_2_SiO_4_F_1.42_(OH)_0.58_ with H_2_O content of ~2 wt%. The electron microprobe analysis was conducted at the Experimental facility in Clermont Ferrand, France. We also characterized topaz using a Horiba Labram HR Evolution Raman Spectrometer. We used a 532 nm laser with a 16 mW of laser power. We collected Raman spectra from 100 – 1300 cm^−1^ and 3000 – 4000 cm^−1^. We note several vibrational modes including modes at 159, 242, 271, 290, 336, 518, 555, 913, 927, 977, and 1156 cm^−1^. In the spectral range of 3000 – 4000 cm^−1^ we note two distinct O-H stretching modes occurring at 3630 and 3642 cm^−1^ (Supplementary Figure SF1). The Raman Spectra were collected at the Experimental Facility in Clermont-Ferrand, France.

We used Resonant Ultrasound Spectroscopic (RUS) facility at the Earth Materials Laboratory, Department of Earth, Ocean and Atmospheric Sciences, Florida State University to determine the elastic constants of topaz crystal at high temperatures. RUS utilizes mechanical resonance spectrum (MRS) of a crystal to determine full elastic tensor. MRS consists of a set of natural frequencies that are related to the elasticity, density, and the sample geometry (Supplementary Figure SF2**)**. A list of observed and calculated sample resonance frequencies, $\frac{df}{d{C}_{ij}}$ and the mode classification is reported in the supplementary section (Supplementary Table ST1a). The temperature dependence of the modes is reported in supplementary section (Supplementary Table ST1b-c). We placed the topaz crystal between two buffer rods 30 cm long and with a 3 mm diameter. The free end of each buffer rod was firmly attached to a lead zirconate titanate (PZT) piezoelectric transducer. One of the transducers was excited with a sweeping sinusoidal signal from DS345 function generator, while the other transducer was connected to SR844 lock-in amplifier that monitors the vibration response of topaz **(**Supplementary Figure SF3). We placed a thermocouple near the sample to record the temperature (Supplementary Figure SF3). We enclosed the topaz crystal and the surrounding ceramic framework in a fused quartz tube and inserted them into the high-temperature furnace (Carbolite MTF 12/28/250). We maintained the temperature within ± 1 K using a proportional-integral-derivative (PID) controller. We heated and cooled the topaz single crystal several times. In the first cycle of heating and cooling, we had placed the topaz single crystal between two alumina buffer rods. After the heating and cooling cycle we noticed that the single crystal of topaz was mechanically damaged and hence we discarded all the RUS results from the first cycle of heating and cooling. We conducted the subsequent heating and cooling cycles by placing the topaz crystals between fused quartz buffer rods. We conducted two additional heating and cooling cycles and checked the single crystal of topaz before and after heating and noticed no mechanical damage (Supplementary Figure SF4).

In RUS method, the topaz crystal is excited with an excitation frequency ($f$) and as it matches with the natural frequency (${{f}_{i}}^{0}$), we observe a resonance. The subscript $i$ denotes the $i\,$^th^ natural frequency of topaz crystal. The vibration is amplified by the quality factor of the resonances ($Q\,=\,\frac{{{f}_{i}}^{0}}{{\rm{\Delta }}f}$), where ${\rm{\Delta }}f$ is the full width at half maxima for the frequency vs. amplitude. The average acoustic energy loss for the resonance modes, ${Q}_{av}^{-1}$ is very low ($20-50\times {10}^{-5}$) indicating low acoustic dissipation (Supplementary Figure SF5). The full elastic constant tensor is determined by comparing the observed resonance frequencies (${f}_{i}^{obs}$) with the calculated resonance frequencies ($\,{f}_{i}^{calc}$) using a least squares method and minimizing the residual, $\Delta F=\sqrt{\frac{1}{N}\sum _{i=1}^{N}{(\frac{{f}_{i}^{obs}-{f}_{i}^{calc}}{{f}_{i}^{calc}})}^{2}}\times 100\, \%$. The $\,{f}_{i}^{calc}$ are calculated using the initial guesstimates of full elastic constant tensor, density, and sample geometry. We used the Rayleigh-Ritz method^14–19^ and the basis functions used in the algorithm^20^ are expressed in powers of Cartesian coordinates^20,21^. The order of polynomial were limited to N = 12, yielding 1365 basis functions which approximates the displacement vector^20^.

**Figure 1:**
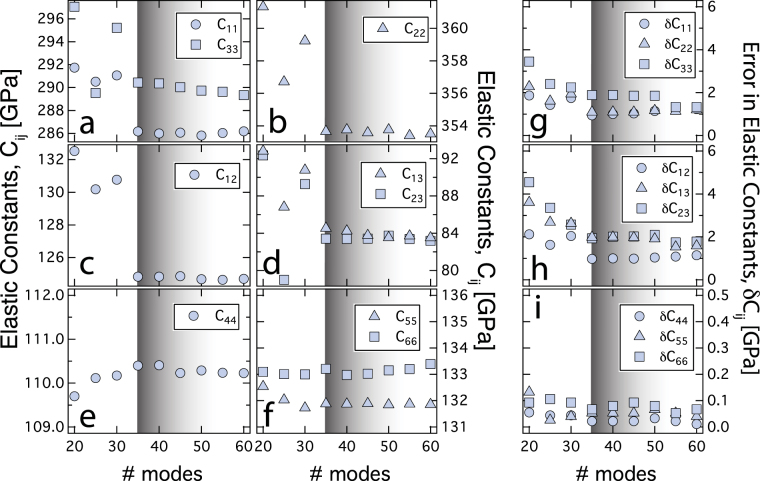
Convergence test and Error analysis. Variation of elastic constants (**a**) C_11_ and C_33_, (**b**) C_22_, (**c**) C_12_, (**d**) C_13_ and C_23_, (**e**) C_44_, (**f**) C_55_ and C_66_, and the errors associated with the components of the elastic constants (**g**) δC_11_, δC_22_, and δC_33_, (**b**) δC_12_, δC_13_, and δC_23_, (**c**) δC_44_, δC_55_, and δC_66_, as the number of modes frequencies are varied in non-linear least square fitting. All these elastic constants are for room temperatures i.e., 293 K. Grey shaded area indicates the least number of modes required to have converged elastic constants and errors. Convergence test and error analysis at higher temperature (~973 K) requires > 40 resonance modes for convergence (Supplementary Figure SF6).

In this study, the temperature range explored is between ~ 293 – 973 K. We collected the MRS spectra between 0.8 – 3.0 MHz and observed ~ 60 resonance modes (Supplementary Table ST1a). In our non-linear least square fits, we varied the number of resonance modes from 20 to 60. We noticed that the components of the full elastic constant tensors vary significantly if the number of resonance modes considered is less than 35. When more than 35 resonance modes are considered in the determination of the elastic constants, the components of the full elastic constant tensor converge and remain unaffected when additional resonance modes are considered in the non-linear fit. For instance, the principal elastic constants,$\,{C}_{11}$, ${C}_{22}$, and ${C}_{33}\,\,$changes by ~ 2 % whereas the off-diagonal elastic constants,$\,{C}_{12}$, ${C}_{13}$, and ${C}_{23}\,\,$changes by 6 – 10% when the resonance modes considered in the non-linear least square fit are enhanced from 20 to more than 35 modes (Fig. 1). Concomitant with the convergence of the elastic constant, the error associated with the full elastic constant tensor also reduces significantly when more than 35 resonance modes are considered in the non-linear least square fit. For instance, the errors associated with the principal elastic constants,$\,\delta {C}_{11}$, $\delta {C}_{22}$, and $\delta {C}_{33}\,\,$are reduced by ~ 50 % whereas the off-diagonal elastic constants,$\,\delta {C}_{12}$, $\delta {C}_{13}$, and $\delta {C}_{23}\,\,$are reduced by 45 – 60 % (Fig. 1). However, the convergence of elastic constants may require additional resonance modes at higher temperature (Supplementary Figure SF6). Hence in our study across the temperature range we have included 55 resonance modes to determine the elastic constants. The residual $\Delta F$ increases from 0.26 to 0.30 as the number of resonance modes considered in the non-linear fit is increased from 20 to 35 (Supplementary Figure SF6). When additional resonance modes (> 35) are considered, $\Delta F$ decreases to 0.28. Although the residual $\Delta F$ varies, the behavior is less sensitive to the variation of resonance modes and does not provide direct clue towards the required number of modes for converged elastic constants and the associated errors. We used the linear thermal expansion coefficients^22^ ${\alpha }_{a}=6.4\times {10}^{-5}\,{K}^{-1}$, ${\alpha }_{b}=5.5\times {10}^{-5}\,{K}^{-1}$, ${\alpha }_{c}=8.1\times {10}^{-5}\,{K}^{-1}$ along [100], [010] and [001] directions to determine the density of the topaz crystal at elevated temperatures. We estimated the uncertainty in elastic moduli ($\delta {C}_{ij}$) from ${[\frac{d{f}_{k}}{d{C}_{ij}}]}_{n\times m}{[\delta {C}_{ij}]}_{m}={[{f}_{k}^{obs}-{f}_{k}^{calc}]}_{n}$ where, the indices $m$ and $n$ represents the number of independent elastic constants (${C}_{ij}$) and the number of resonance frequencies respectively. We also determined the compliance tensor $[{S}_{ij}]$, which is related to the inverse of the full elastic constant tensor ${[{C}_{ij}]}^{-1}$.

## Results

**Figure 2:**
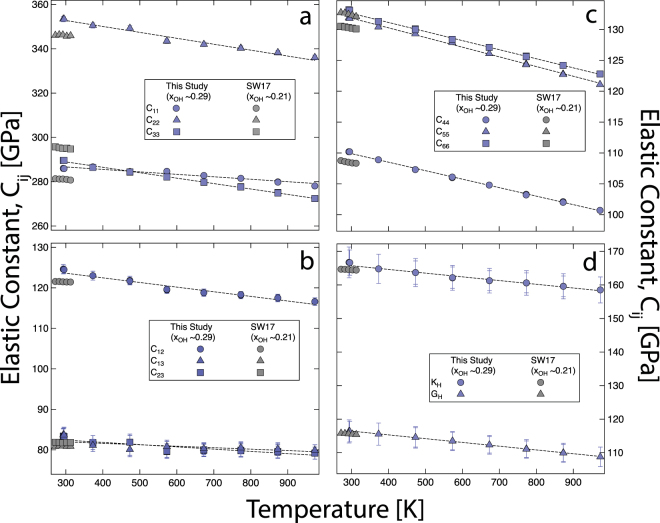
Temperature dependence of full elastic constant tensor. **(a)** Principal components (${C}_{11},\,{C}_{22},\,{C}_{33}$) **(b)** off-diagonal components (${C}_{12},\,{C}_{13},\,{C}_{23}$), **(c)** shear elastic constants (${C}_{44},\,{C}_{55},\,{C}_{66}$), and **(d)** the aggregate elastic moduli (${K}_{H}$ and ${G}_{H}$) of topaz as a function of temperature. The filled grey symbols are from recent RUS experiments on topaz crystal^13^ with ${x}_{OH}=\mathrm{0.21.}\,\,$The dashed lines represent the linear fits $\frac{d{C}_{ij}}{dT}$, $\frac{d{K}_{H}}{dT}$, and $\frac{d{G}_{H}}{dT}$.

We compared room temperature (297 K) elastic constants of the topaz sample measured in our study with the elastic constants of topaz reported in previous studies (Table 1). The principal elastic constants (${C}_{11}$, ${C}_{22}$ and ${C}_{33}$) and the shear elastic constants (${C}_{44}$, ${C}_{55}$ and ${C}_{66}$) from this study are in good agreement with the previous experimental measurements^10,13,23,24^ within 4%. The measured elastic constants ${C}_{11}$, ${C}_{22}$ and ${C}_{33}$ also compare well with recent *first principles* simulations on topaz-OH^11,12^. The shear elastic constants ${C}_{44}$, ${C}_{55}$, and ${C}_{66}$ agrees with previous experimental results at room temperature^13^ (Fig. 2, Supplementary Table ST2a). The shear- elastic constants however, differ from the *first principles* calculations^11^ by 4%, 13%, and 8% respectively. This is likely related to (a) difference in the temperature, i.e., *first principles* simulations were at static conditions (0 K) whereas, our results are at room temperatures, (b) the *first principles* results were for the topaz-OH, i.e., hydroxyl end member whereas our study is conducted on a natural crystal of topaz with enrichment in the fluorine content with a stoichiometry of Al_2_SiO_4_F_1.42_(OH)_0.58_ and (c), *first principles* methods often uses approximations that underestimates or overestimates the elastic constant by few percent but often bracket experimental results. For the off-diagonal components of the elastic tensor, we observed a relatively significant difference between ${C}_{22}$, ${C}_{13}$ and ${C}_{23}$ with the previous studies, except ${C}_{12}$ only differs by 3%. We also note that the error estimate of the ${C}_{23}$, ${C}_{13}$, and ${C}_{12}$ are relatively larger than the error estimate of the principal and shear elastic constants.

**Table 1:** Room temperature elastic constant tensor of topaz from the present study compared with the previous studies.

Chemical Formula	Method	Elastic Constants [GPa]									*Ref*.
		C_11_	C_22_	C_33_	C_23_	C_13_	C_12_	C_44_	C_55_	C_66_	
Al_2_SiO_4_F_2_	Plate Resonance	278.8	344.8	292.3	80.3	80.6	120.4	108.6	132.9	130.3	^10^
Al_2_SiO_4_F_1.56_(OH)_0.42_	RUS-Sphere	281.2	346.2	295.0	81.8	80.9	121.5	108.5	132.5	130.3	^13^
Al_2_SiO_4_F_1.42_(OH)_0.58_	RUS-RPP	286.0	353.4	289.6	83.4	83.7	124.6	110.2	131.9	133.2	*
Al_2_SiO_4_(OH)_2_	*first principles*	285.6	357.3	289.2	87.9	76.9	122.0	105.5	114.6	122.5	^11^

*This study, errors (δ) for C_11_, C_22_, C_33_, C_23_, C_13_, C_12_, C_44_, C_55_, C_66_ are 1.2, 1,2, 1.3, 1.8, 1.6, 1.1, 0.0, 0.0, 0.1 GPa respectively.

We analyzed the axial compressibilities ($${\beta }_{i}$$, where *i* is the crystallographic axis) of topaz, where $${\beta }_{a}=$$$${S}_{11}+{S}_{12}+{S}_{13}$$, $$\,{\beta }_{b}={S}_{21}+{S}_{22}+{S}_{23}$$, and $$\,{\beta }_{c}={S}_{31}+{S}_{32}+{S}_{33}$$ represents the compressibility along the $$a$$, $$b$$, and $$c$$ axes respectively. We used the components of the $${C}_{ij}$$ and $${S}_{ij}$$ tensors to calculate the aggregate elastic moduli of topaz following Voight-Reuss-Hill (VRH) approximation^24,25^. We calculated compressional ($${V}_{P})$$ and shear ($${V}_{S})$$ sound velocities using the Hill averaged bulk modulus ($${K}_{H}$$), shear modulus ($${G}_{H})$$ and the density ($$\rho $$) (Supplementary Table ST2b).

As a function of temperature, we observe a linear decrease in all the individual components of the full elastic moduli tensor (${C}_{ij}$) (Fig. 1). Temperature derivatives of elastic constants $(\frac{d{C}_{ij}}{dT}$, $\frac{dG}{dT}$, $\frac{d{K}_{S}}{dT}$, and $\frac{d{K}_{T}}{dT})$, sound velocities $(\frac{d{V}_{P}}{dT}$ and $\frac{{dV}_{S}}{dT})$, and compressibility $(\frac{d{\beta }_{V}}{dT},\frac{d{\beta }_{i}}{dT})\,\,$where the subscript “${\beta }_{V}$” and “${\beta }_{i}$” refers to volume and axial compressibilities with *i* = *a*, *b*, and *c* directions (Supplementary Table ST2c). We estimated the Debye temperature of topaz using the equation ${{\rm{\Theta }}}_{D}=\frac{h}{k}{(\frac{3}{4\pi {V}_{a}})}^{\frac{1}{3}}{V}_{m}$, where, ${V}_{m}={[\frac{1}{3}(\frac{2}{{{V}_{S}}^{3}}+\frac{1}{{{V}_{P}}^{3}})]}^{-\frac{1}{3}}$ is the mean sound velocity and is related to the compressional (${V}_{P}$) and shear (${V}_{S}$) velocity, $h$ is the Plank constant, $k$ is the Boltzmann constant, ${V}_{a}=\frac{M}{n{N}_{A}\rho }\,\mathrm{is}\,\mathrm{average}\,\mathrm{atomic}\,\mathrm{volume},\,{N}_{A}$ is the Avogadro number, $\rho$ is density, $M$ is molar mass, and $n$ is the number of atoms^26–28^. Our estimate of the Debye temperature (${{\rm{\Theta }}}_{D}$) of topaz at room temperature is ~ 910 K. We also estimated the Grüneisen parameter, $\gamma ={\alpha }_{V}\frac{{K}_{S}}{\rho {C}_{P}}={\alpha }_{V}\frac{{K}_{T}}{\rho {C}_{V}}$, where, ${\alpha }_{V}$ is volumetric thermal expansion coefficient, ${K}_{S}$ is the adiabatic bulk moduli, ${K}_{T}$ is the isothermal bulk moduli, ${C}_{P}$ is the heat capacity at constant pressure, and ${C}_{V}$ is the heat capacity at constant volume. To estimate $\gamma$, we used ${K}_{S}={K}_{H}$ (averaged bulk modulus), we used ${\alpha }_{V}\,\,$and ${C}_{P}$ from previous experimental studies^22,29,30^. We used the $\gamma$ and ${\alpha }_{V}$ and determined ${K}_{T}$ **(**Supplementary Table ST2b**)**. We used ${K}_{S}$, ${K}_{T}$ to calculate the bulk adiabatic (${\beta }_{S}$) and isothermal (${\beta }_{T}$) compressibility of topaz ${\beta }_{x}=\frac{1}{{K}_{x}}$, where $x=\{S,T\}$ and their temperature derivatives (Supplementary Table ST2b).

We determined the sound velocities $${V}_{P}(\hat{n})$$ and $$\,{V}_{S}(\hat{n})$$ of topaz as a function of propagating direction ($$\hat{n}$$) by solving the Christoffel equations^31^. Shear waves propagating in the crystal are affected by the propagation direction as well as the direction of particle displacement (polarization). This gives us the fastest ($${V}_{S1}$$) and slowest ($${V}_{S2})$$ shear wave velocities. We used the maximum ($${{V}_{x}}^{max}$$) and minimum ($${{V}_{x}}^{min}$$) wave velocities to calculate the anisotropy $$A{V}_{x}=\frac{{{V}_{x}}^{max}-{{V}_{x}}^{min}}{\frac{1}{2}({{V}_{x}}^{max}+{{V}_{x}}^{min})}\times 100\, \% $$, where $$x=\{P,S1,S2\}$$. We calculated the shear wave polarization anisotropy at each direction by $$A{V}_{S}=\frac{{V}_{S1}-{V}_{S2}}{\frac{1}{2}({V}_{S1}+{V}_{S2})}\times 100\, \% $$.

At room temperatures, we note that the fastest P-wave velocity, ${{V}_{P}}^{max}=10.16\,km/s$ is along a direction that is inclined to the *b*-axis by $\sim 30^\circ \,\,$and lies in the (001) plane. The slowest P-wave velocity, ${{V}_{P}}^{min}=9.01\,km/s$ is along the *a*-axis. Our observation of the fastest P-wave velocity, ${{V}_{P}}^{max}$ along the ~*b*-axis and the slowest ${{V}_{P}}^{min}$ along the *a*-axis is consistent with the fact that the principal components bear the relationship ${C}_{22}\gg {C}_{33}\ge$ ${C}_{11}.$ We note that the maximum shear wave velocities for the S1 component i.e., ${{V}_{S1}}^{max}=\,6.15\,km/s$ is along the *a-*direction whereas the maximum S2 shear wave velocity, ${{V}_{S2}}^{max}=\,6.12\,km/s$ is along the *a*-direction. The minimum S1-wave velocity, ${{V}_{S1}}^{min}=\,5.69\,km/s$ lies in the (001) plane and is oriented $\approx 22^\circ \,\,$with the *b*-axis, the minimum S2- wave velocity, ${V}_{S2}=5.23\,km/s$. The compressional wave azimuthal anisotropy, $A{V}_{P}$ and shear wave polarization anisotropy $A{V}_{S}$ for Al_2_SiO_4_F_1.42_(OH)_0.58_ are $\sim 11.9\, \%$, and $\sim 12.9\, \%$ respectively (Supplementary Figure SF7).

## Discussion

The elasticity and the elastic anisotropy of topaz can be understood in terms of the crystal structure. Topaz crystal structure consists of SiO_4_ tetrahedral units sharing the corners with the edge shared pairs of AlO_4_(F,OH)_2_ octahedral units (Supplementary Figure 1). The compressibility of AlO_4_(F,OH)_2_ units in the crystal structure are greater than the SiO_4_ units^11,22,32^. The alternating of AlO_4_(F,OH)_2_ and SiO_4_ units in along the *c*-axes results in “weak zones” or cleavages parallel to the *a-b* plane, i.e., (001) and perpendicular to the *c*-direction^32–34^ (Supplementary Figure 1).

We observed a significantly high stiffness along *b*-axis, i.e., [010] direction of the crystal structure, indicated by the relationship $\,{C}_{22}\gg {C}_{33}\ge$ ${C}_{11}$. This is translated to a significantly high compressibility observed along *a-* and *c-* axes compared to the compressibility along *b-* axis, with the relation, *β*_a_ = *1.34* × *β*_b_ and *β*_c_ = *1.60* × *β*_b_. The anisotropy in axial compressibility is likely to be related to the of higher compressibility of the “weak zones” consisting only of AlO_4_(F,OH)_2_ units.

The sound velocity anisotropy $A{V}_{P}=11.9\, \%$ is smaller than the previous experimental study $A{V}_{P}$(topaz-F)^10^ $\mathrm{by}\, \sim 0.8\, \%$ but it is greater than the $A{V}_{P}$ (topaz-OH)^11^ determined by *first principles* simulations by $\sim 3.1\, \%$. The maximum polarization anisotropy of shear wave velocity is smaller than the previous experimental study $A{V}_{S}\,$(topaz-F)^10^ by 6 %, than the $A{V}_{S}\,$(topaz-OH)^11^ determined by *first principles* simulations by ~ $34.9\, \%$. This could be due to several factors including the difference in the OH-F content, approximations made in the *first principles* simulation and difference in the room temperature experiments and static conditions for the simulation. When compared to other hydrous aluminosilicate minerals and major mantle mineral phases, topaz showed a P-wave anisotropy ($A{V}_{P}\,{\rm{ \% }}$) $\sim 10\, \%$ lower than that of andalusite, diaspore, olivine, and quartz (Supplementary Figure SF8). However, compared to the dense mineral phase corundum, $A{V}_{P}$ of topaz is $\sim 30\, \%$ greater. S-wave anisotropy ($A{V}_{S}\,{\rm{ \% }}$) of aluminosilicate phases show wide variation with quartz being the greatest followed by corundum. This is followed by the S-Wave anisotropy of topaz, which is very similar to that of andalusite, and diaspore (Supplementary Figure 8). The $A{V}_{S}$ of topaz is slightly lower than that of olivine ($A{V}_{S}\approx 18\,{\rm{ \% }}$).

**Figure 3:**
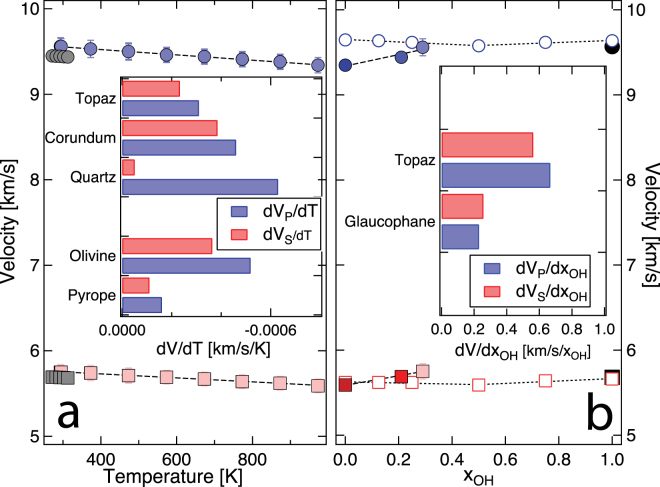
Compressional (${V}_{P}$) and shear (${V}_{S}$) wave velocity as a function of temperature and composition. **(a)** ${V}_{P}\,$(light blue filled circles) and ${V}_{S}$ (light red filled squares) wave velocities in topaz as a function of temperature. The inset shows the temperature derivatives of wave velocities, $\frac{d{V}_{P}}{dT}\,$(blue) and $\frac{d{V}_{S}}{dT}$ (red) for topaz and other relevant mantle mineral phases such as corundum^37^, quartz^35^, olivine^38^, and pyrope^39^. **(b)** Variation of ${V}_{P}$ (blue filled circles) and ${V}_{S}$ (red filled squares) of topaz as a function of composition, i.e., $({x}_{OH}=\frac{{C}_{OH}}{{C}_{OH}+{C}_{F}})$ where ${C}_{OH}$ and ${C}_{F}$ refers to the OH and F content in topaz. Solid symbols represent sound velocities of fluorine end-member^10^, OH end member^11^, and the composition of ${x}_{OH}=0.21$^13^. Open symbols represent *first principles* simulations with varying OH and F content^12^. The inset shows the compositional dependence of ${V}_{P}\,\,$and ${V}_{S}$ wave velocities i.e., $(\frac{d{V}_{P}}{d{x}_{OH}})$ (blue) and $(\frac{d{V}_{S}}{d{x}_{OH}})$ (red) for topaz (*from this study*) and a *first principles* based study on glaucophane amphibole^40,41^.

The temperature derivate of single crystal elastic coefficients ($\frac{d{C}_{ij}}{dT}$) from the present study agrees very well with previous experiments^10^. The slight discrepancy in the individual $\frac{d{C}_{ij}}{dT}$ between the present study and the previous results remains unexplained since the sampling density of elasticity and the range of temperature explored in previous experimental study remains unreported^10^. The temperature derivative i.e., $\frac{d{V}_{P}}{dT}$, and $\frac{d{V}_{S}}{dT}$ of topaz from the present study are −3.10 and −2.30 $\times$ 10^−4^ km/s/K respectively (Fig. 3). The P-wave temperature derivative $\frac{d{V}_{P}}{dT}$ of aluminosilicates and major mantle phase vary as quartz < olivine < corundum < topaz < pyrope, i.e., the aggregate longitudinal sound wave velocity for pyrope is least temperature sensitive. Similarly, the S-wave temperature derivative $\frac{d{V}_{S}}{dT}$ of aluminosilicates and major mantle phase exhibits the following trend: corundum < olivine < topaz < pyrope < quartz, i.e., the aggregate shear sound wave velocity for quartz is least temperature sensitive. However, quartz undergoes a temperature dependent displacive transition where the temperature derivatives of the sound wave velocity show significant non-linear behavior in the vicinity of the transition ~ 800 – 900 K^35^. Hence, the temperature derivatives of sound wave velocity for topaz lies somewhat in-between the various aluminosilicates and mantle phases. In contrast to the temperature derivatives, the pressure derivatives of the sound wave velocity are significantly larger and have been estimated using *first principles* simulations for OH end member and are of the order of ~0.07 and 0.02 km/s/GPa for $\frac{d{V}_{P}}{dP}$, and $\frac{d{V}_{S}}{dP}$ respectively^11^.

The sound wave velocity is also sensitive to the chemistry of topaz. In particular, the F and OH content of topaz is known to affect elasticity^12^. However, the effect is very nonlinear, the *first principles* simulations across the F and OH end member of topaz indicate that the sound velocity gradually decreases up to the 50:50 composition. The compositional dependence ${V}_{P}$ and ${V}_{S}$ for $0.0 < {x}_{OH} < 0.5$ can be described with polynomial functions: ${V}_{P}=-0.10{x}_{OH}^{2}-0.09{x}_{OH}+9.65$ km/s and ${V}_{S}=-0.22{x}_{OH}^{2}+0.05{x}_{OH}+5.62\,\mathrm{km}/{\rm{s}}$, i.e., $\frac{d{V}_{P}}{d{x}_{OH}}$ ~ −0.09 km/s and $\frac{d{V}_{S}}{d{x}_{OH}}$ ~ +0.05 km/s. For ${x}_{OH} > 0.5,$ sound velocities can be described by ${V}_{P}=-0.16{x}_{OH}^{2}+0.36{x}_{OH}+9.44$ km/s and ${V}_{S}=-0.24{x}_{OH}^{2}+0.50{x}_{OH}+5.40\,\mathrm{km}/{\rm{s}}$, i.e., $\frac{d{V}_{P}}{d{x}_{OH}}\,$ ~ 0.36 km/s and $\frac{d{V}_{S}}{d{x}_{OH}}\,$ ~ 0.50 km/s. In contrast to the recent *first principles* simulations^12^, the sound velocity fluorine end member topaz determined by plate-resonance technique is significantly softer^10^. The sound velocity of fluorine and OH bearing topaz from the present study also deviates from the *first principles* simulations^11,12^ (Fig. 3). To our knowledge, there are no experimental results on the sound velocity for the OH end member of topaz. The sound velocities for the OH end member from two different *first principles* simulation exhibit good agreement^11,12^. However, these simulations are at static conditions i.e., 0 K, whereas the experimental results are at room temperature (~ 298 – 300 K). Therefore, we applied a static temperature correction to the sound velocities obtained from *first principles* simulations^11,12^ using $\frac{d{V}_{P}}{dT}$ and $\frac{d{V}_{S}}{dT}$ from the present study. We compared thermally corrected *first principles* sound velocity results with experimentally determined sound velocity from previous^10,13^ and present studies (Fig. 3). The compositional dependence of the sound velocity from the experimental studies varies almost linearly as: ${V}_{P}=0.67{x}_{OH}+9.34$ km/s and ${V}_{S}=0.53{x}_{OH}+5.59$ km/s, i.e., $\frac{d{V}_{P}}{d{x}_{OH}} \sim 0.67$ (± 0.22) km/s and $\frac{d{V}_{S}}{d{x}_{OH}}$ ~ $0.53$ (± 0.07) km/s. This indicates that the effect of composition on the sound velocity is 3 – 4 orders of magnitude larger. Based on our analysis, we note that increase in the hydroxyl component enhances the velocity in the composition range of $0.0 < {x}_{OH} < 0.3$. This is likely to have implications for high pressures where topaz tends to incorporate OH component as observed in ultra high pressure metamorphic rocks^2,5^. The estimate for the compositional derivative of the velocity could be significantly improved by having more results across the F and OH end members. However, care must be taken to interpret the results since elastic constants determined using RUS is sensitive to various factors including the geometry of the single crystal, the number of resonance modes used in the non-linear least square fit, and surface imperfection. So care must be taken when interpreting the effect of composition on the sound velocity. We examined the effect of resonance mode on the elasticity and sound velocity (Fig. 1) and noticed that elasticity and sound velocity determination for topaz with more than 55 resonance mode are adequate. Based on the effect of temperature, pressure, and chemistry, we note that, 0.1 increment in ${x}_{OH}$ will cause opposite but similar magnitude change in $d{V}_{P}$ as ~ 216 K and similar effect of ~ 1.3 GPa of pressure.

**Figure 4:**
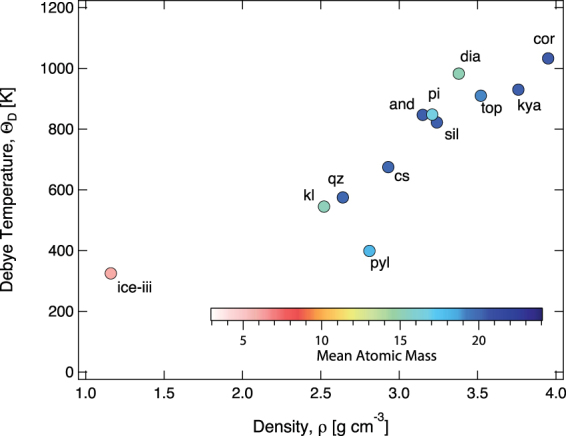
Variation of Debye temperature with density for mineral phases in Al_2_O_3_-SiO_2_-H_2_O (ASH) ternary. The Debye temperature shows strong correlation with density of mineral phases The symbols are colored based on the mean atomic mass ($\bar{M}$) of each mineral phase (for further details please refer to Table 2, Supplementary Figure SF8).

In order to understand how the physical properties such as sound velocity vary across the mineral phases in hydrated subducting sediments, we examined sound velocity and anisotropy of mineral phases in the Al_2_O_3_-SiO_2_-H_2_O ternary system, which includes andalusite, diaspore, kaolinite, quartz, phase-pi, pyrophyllite, and topaz. (Fig. 4, Table 2). The Debye elastic temperature (${{\rm{\Theta }}}_{D}$) influences the phase equilibria through the Clapeyron slopes^36^ and hence it is important to gain an in-depth understanding of how Debye temperature is affected as a function of density and mineral compositions. We note that the Debye temperature (${{\rm{\Theta }}}_{D}$) varies almost linearly with the density and inversely with the mean atomic mass (Fig. 4). This is consistent with the observations in major mantle mineral phases^36^.

**Table 2:** Density (*ρ*), mean atomic mass $(\bar{M})$, sound velocities (*V*_*P*_, *V*_*S*_) and Debye temperature (*Θ*_*D*_) of mineral phases of the Al_2_O_3_-SiO_2_-H_2_O (ASH) ternary system.

Mineral	Abbreviation	Stoichiometry	ρ [g/cm^3^]	$\bar{{\boldsymbol{M}}}$	V_P_ [km/s]	V_S_ [km/s]	Θ_D_ [K]	Reference
Corundum	cor	Al_2_O_3_	3.95	20.4	10.88	6.40	1032	^37^
Quartz	qz	SiO_2_	2.64	20.0	6.09	4.12	575	^35^
Coesite	cs	SiO_2_	2.93	20.0	8.17	4.58	675	^42^
Ice	ice-iii	H_2_O	1.16	6.0	3.66	2.01	326	^43^
Andalusite	and	Al_2_SiO_5_	3.15	20.3	9.76	5.65	848	^44^
Sillimanite	sil	Al_2_SiO_5_	3.24	20.3	9.65	5.42	823	^45^
Kyanite	kya	Al_2_SiO_5_	3.76	20.3	9.68	5.87	930	^45^
Diaspore	dia	AlOOH	3.38	12.0	9.42	5.83	984	^46^
Kaolinite	kl	Al_2_Si_2_O_5_(OH)_4_	2.52	15.2	6.23	3.55	545	^47^
Pyrophyllite	pyl	Al_2_Si_4_O_10_(OH)_2_	2.81	18.0	3.61	2.76	400	^48^
Phase-Pi	pi	Al_3_Si_2_O_7_(OH)_3_	3.21	16.7	8.86	5.28	849	^40^
Topaz	top	Al_2_SiO_4_F_1.42_(OH)_0.58_	3.52	19.1	9.56	5.75	910	*this study*

## Conclusions

We investigated the elasticity of natural single crystal topaz with a stoichiometry of Al_2_SiO_4_F_1.42_(OH)_0.58_ using resonant ultrasound spectroscopy method. We determined the temperature dependence of full elastic tensor, aggregate elastic moduli, compressibility, Grüneisen parameter, and sound velocities of topaz in the temperature range of 297 – 973 K. In topaz, the sound velocity varies as a function temperature and composition i.e., OH and F content. We note that topaz has one of the lowest temperature derivatives among aluminosilicates and other major mantle phases. Elasticity results indicate that topaz is quite anisotropic and comparable with other mantle phases and aluminosilicate minerals. We also note that the sound velocity, Debye temperature of mineral phases vary as a function of density in the hydrous aluminosilicates phases belonging to the Al_2_O_3_-SiO_2_-H_2_O ternary system that are relevant for the hydrated subducted sediments.

### Data availability

All data generated or analyzed during this study are included in this published article and its Supplementary Information files.

## Electronic supplementary material

Supplementary Information and Figures

Supplementary Tables ST1–4
