# Supplementary material for: Single crystal elasticity of natural topaz at high-temperatures
Source: Sci Rep. 2018 Jan 22;8:1372. doi: 10.1038/s41598-017-17856-3 (PMC5778148; doi:10.1038/s41598-017-17856-3)
Supplement: Supplementary file 1 — Supplementary Information and Figures [file 41598_2017_17856_MOESM1_ESM.pdf]

01/04/2018

Single crystal elasticity of natural topaz at high-temperatures

Sumudu Tennakoon[1*], Ye Peng[1], Mainak Mookherjee[1#], Sergio Speziale[2], Geeth Manthilake[3], Tiglet Besara[4], Luis Andreu[1], Fernando Rivera[1]

[1]Earth Materials Laboratory, Department of Earth, Ocean and Atmospheric Sciences, Florida State University, Tallahassee, FL 32306, USA.

*stennakoon@fsu.edu, #mmookherjee@fsu.edu

[2] GFZ German Research Centre for Geosciences, 14473 Potsdam, Germany

[3]Université Clermont Auvergne, CNRS, IRD, OPGC, Laboratoire Magmas et Volcans, F-63000 Clermont-Ferrand, France

[4]National High Magnetic Field Laboratory, Tallahassee, FL 32310, USA.

A. Supplementary Figure Captions
B. Supplementary Figures, SF1-SF7
C. Supplementary Tables, ST1-ST4

A. Supplementary Figure Captions

Figure SF1. Raman spectra of the single crystal topaz at room temperature: **(a)** 100-1300 cm^{-1} and **(b)** 3000-4000 cm^{-1} regions show two distinct O-H stretching modes. The green refers to the background and the blue curve is the peak fit. The OH stretching region is deconvolved in to two peaks with peak center at 3630 cm^{-1} and 3642 cm^{-1}. The residuals are also shown with filled circles at the top. The inset shows the single crystal diffraction pattern along (0kl), (h0l), and (hk0) planes. (**c**) The crystal

structure of topaz from the refinement of the single crystal X-Ray diffraction data is shown. Since the precise hydrogen positions were not refined, the fluorine (F) and the hydroxyl anion (OH) are shown together and indicated by the light blue spheres.

Figure SF2. (a) Mechanical resonance spectrum (MRS) collected at room temperature for the topaz crystal. The frequency range over which, the MRS is collected ranges up to 3.1 MHz. The greyed rectangular box with dashed outline in in (**a**) is zoomed in figure (**b**) Also shown are the mode displacement plots for selected resonance modes. (**c**) The temperature dependence of the modes are shown in a stacked plot, and the corresponding temperatures are indicated in the right hand side vertical axes.

Figure SF3. (a) High temperature Resonant Ultrasound Spectroscopy (RUS) setup used in this study for the determination of the full elastic constant tensor. The inset shows the single crystal of topaz used in this study. The crystal is placed between two alumina rods that buffer between the crystal and the lithium niobate ($LiNbO_3$) transducers. **(b)** Schematic diagram showing the high temperature RUS setup.

Figure SF4. (a) The topaz single crystal shows a crack. The crystal was mechanically damaged after the first heating and cooling cycle. The crystal was placed between two alumina buffer rods. All resonant mode data were discarded from this run. (b) The topaz single crystal after the second (H-I, C-I) and third heating (H-II) and cooling (C-II) cycle. The crystals were placed within fused quartz buffer rods. The data from the second and third heating and cooling cycle are used for further analysis.

Figure SF5. Plot of average acoustic energy loss Q_{av}^{-1} as a function of heating and cooling cycle.

Figure SF6. Variation of elastic constants (**a**) C_{11} and C_{33}, (**b**) C_{22}, (**c**) C_{12}, (**d**) C_{13} and C_{23}, (**e**) C_{44}, (**f**) C_{55} and C_{66} and the errors associated with the components of the elastic constants (**g**) C_{11}, C_{22}, and C_{33}, (**b**) C_{12}, C_{13}, and C_{23}, (**c**) C_{44}, C_{55}, and C_{66}, as the number of mode frequencies are varied in non-linear least square fitting. All these elastic constants are for high temperatures of 923 K. Grey shaded area indicates the least no of modes required to have converged elastic constants and errors. (j) the residual (ΔF) for 298 K and 723 K and how it varies as the number of mode frequencies are varied in non-linear least square fitting.

Figure SF7. Compressional (V_P) and shear (V_{S1} & V_{S2}) wave velocities, and the polarization anisotropy (AV_S) as a function of propagation direction in minerals from Al_2O_3-SiO_2-H_2O (ASH) ternary system, **(a)** fluorine end-member of topaz[10], (**b)** topaz ($Al_2SiO_4F_{1.42}(OH)_{0.58}$) (this study), (**c)** hydroxyl end-member of topaz[11], **(d)** quartz[35]**, (e)** andalusite[36], **(f)**, corundum[37], and (**g)** diaspore[38].

Figure SF8. Ternary plot showing the (**a)** Density and **(b)** Debye temperature (Θ_D) of mineral phases in the Al_2O_3-SiO_2-H_2O (ASH) system. **(c)** Debye temperature (Θ_D) of mineral phases of the ASH ternary system as a function of density **(Table 1)**.

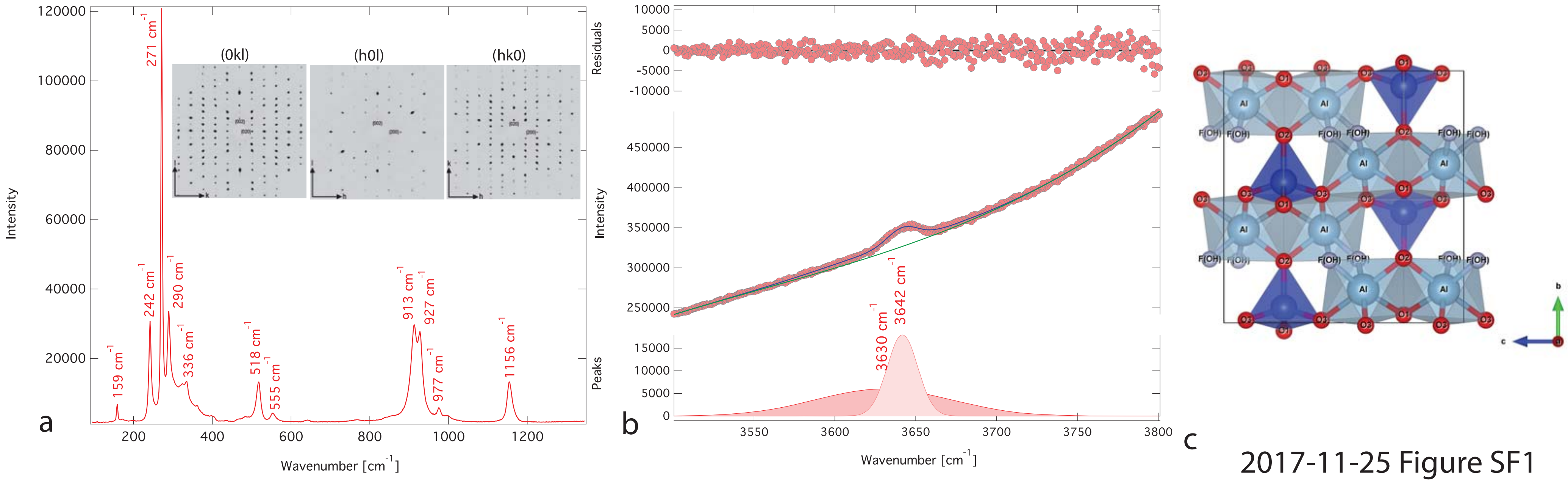

2017-11-25 Figure SF1

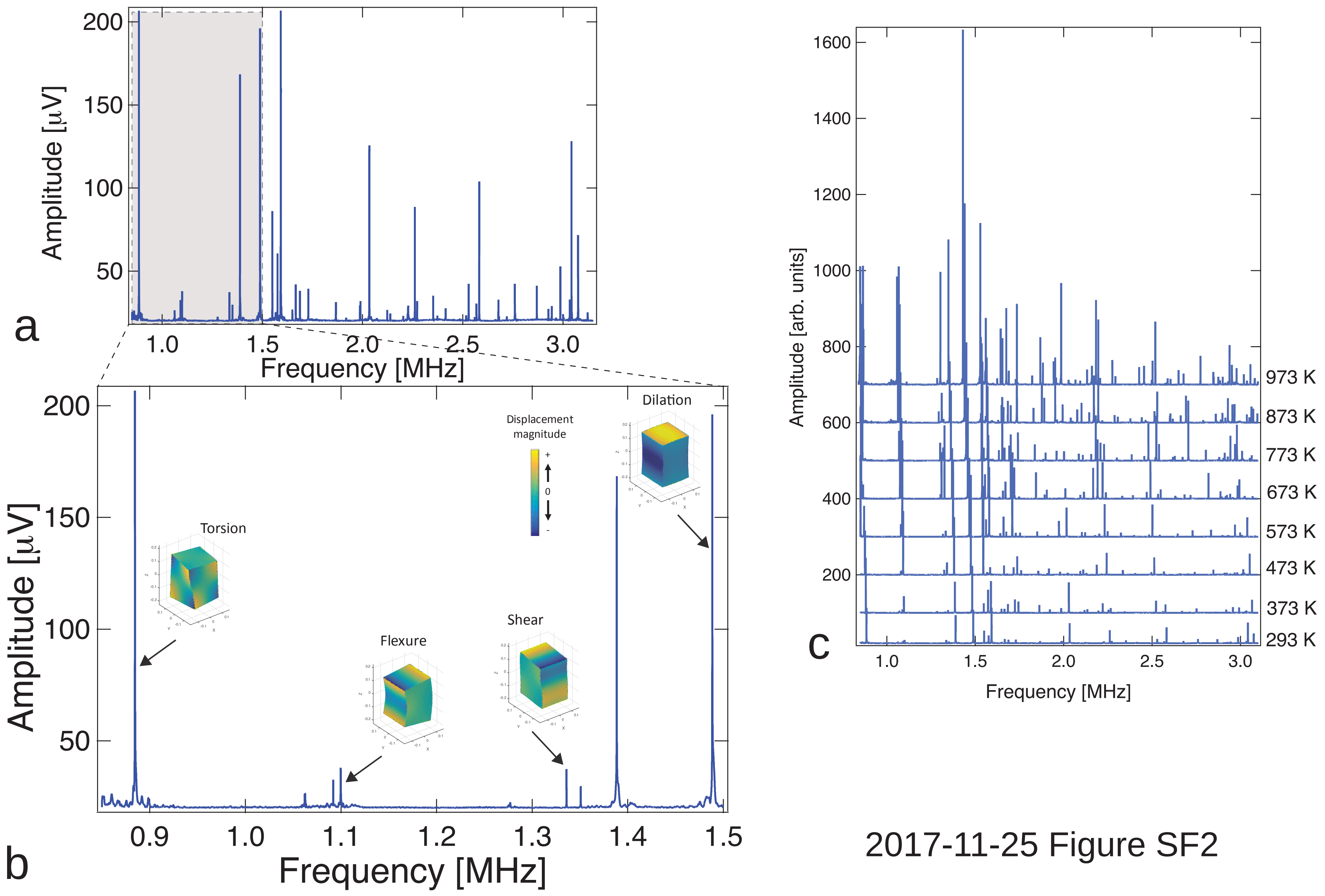

2017-11-25 Figure SF2

a

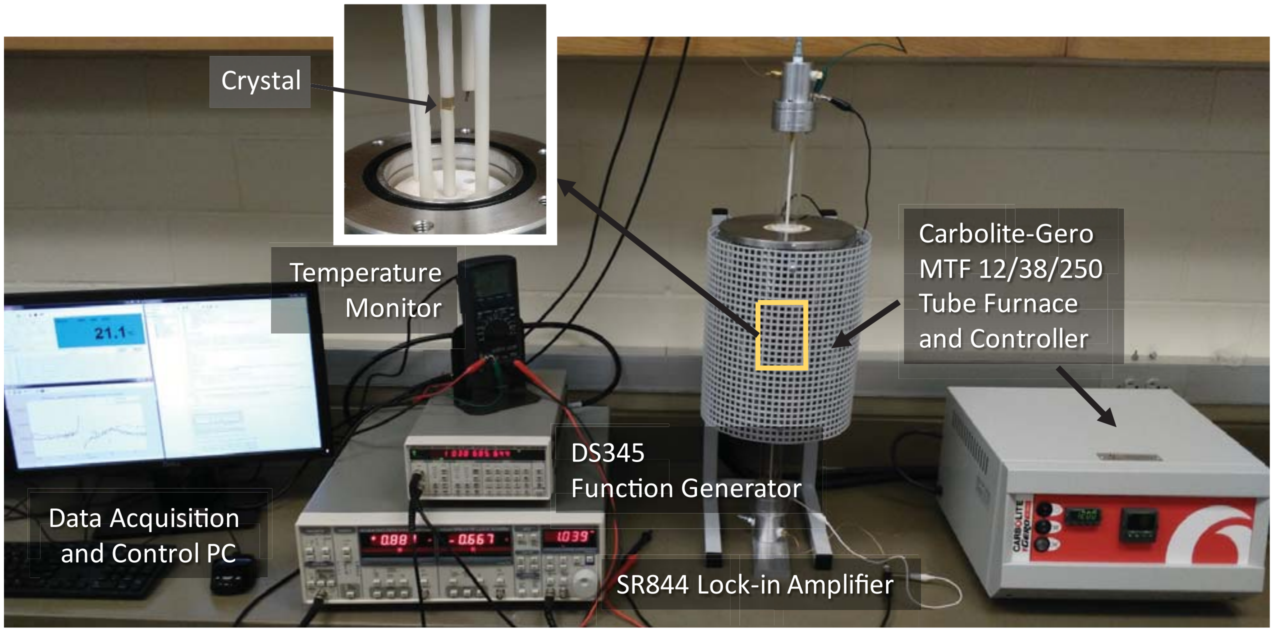

b

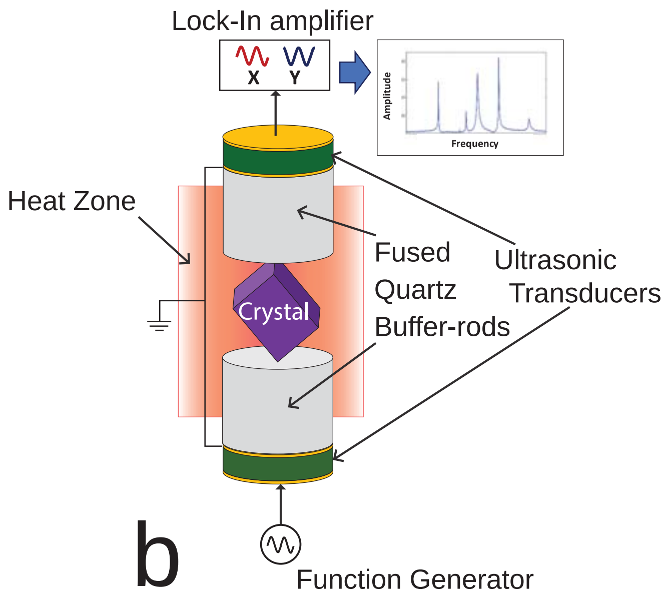

2017-11-25 Figure SF3

a

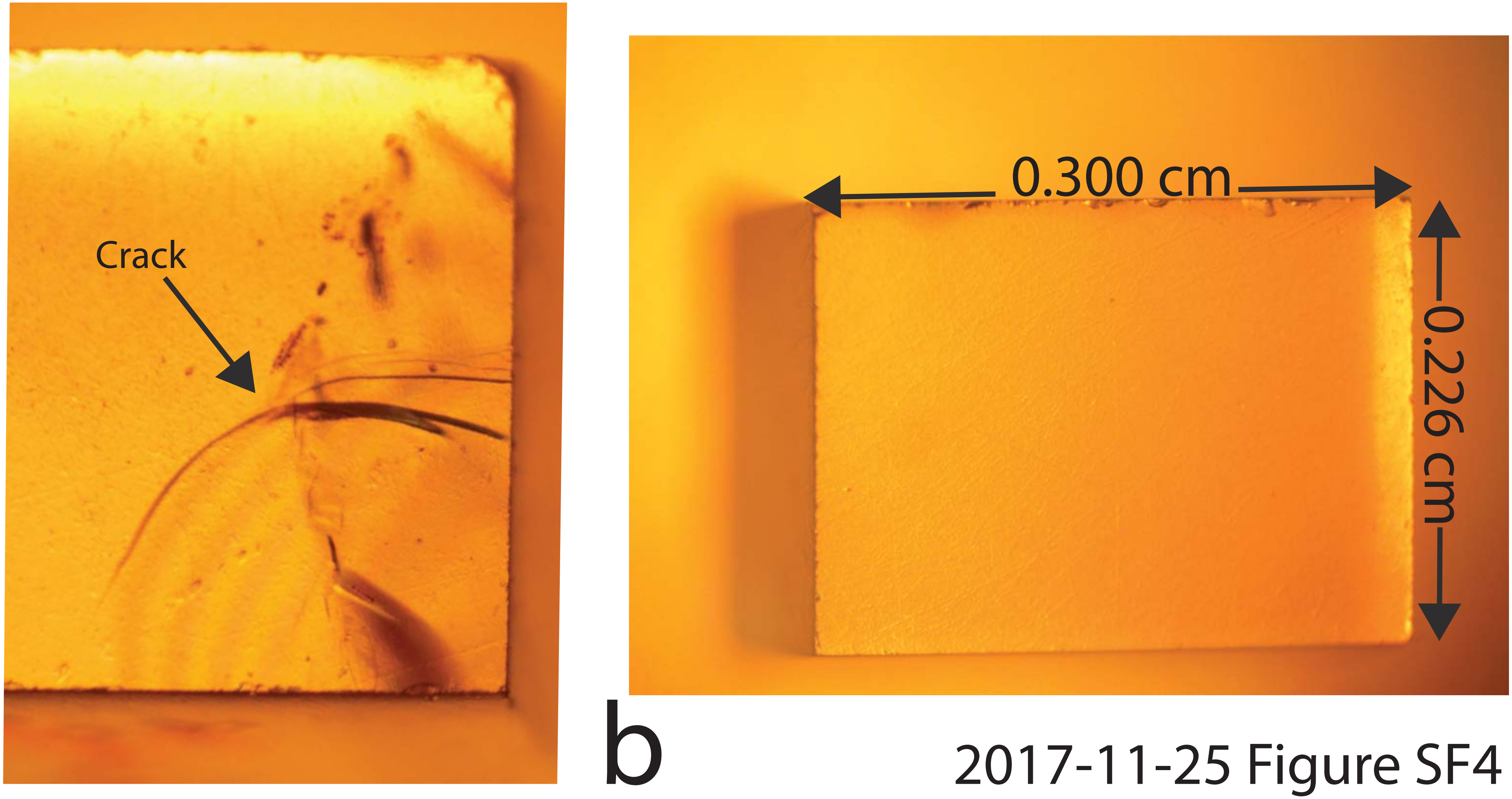

b

2017-11-25 Figure SF4

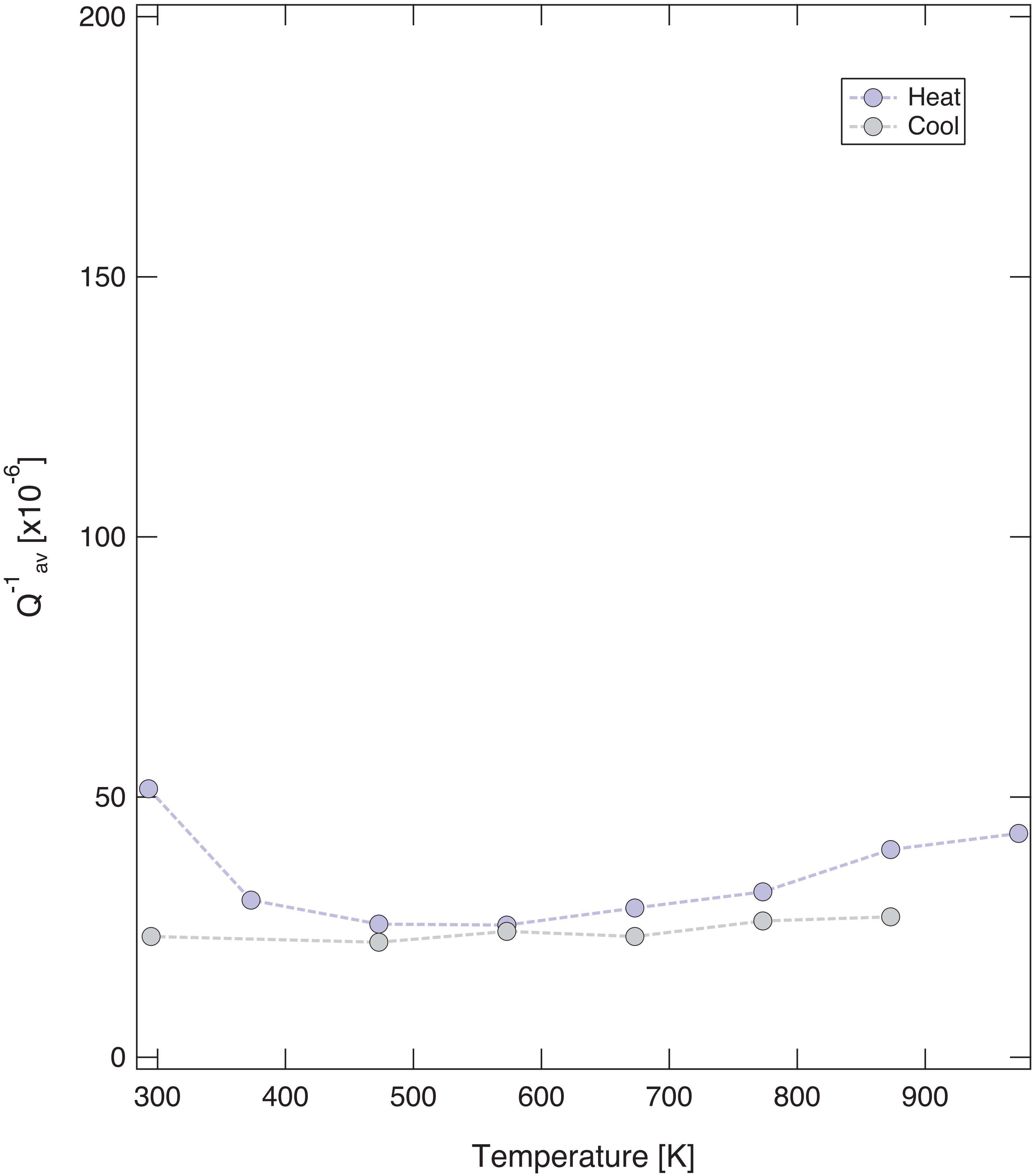

2017-11-25 Figure SF5

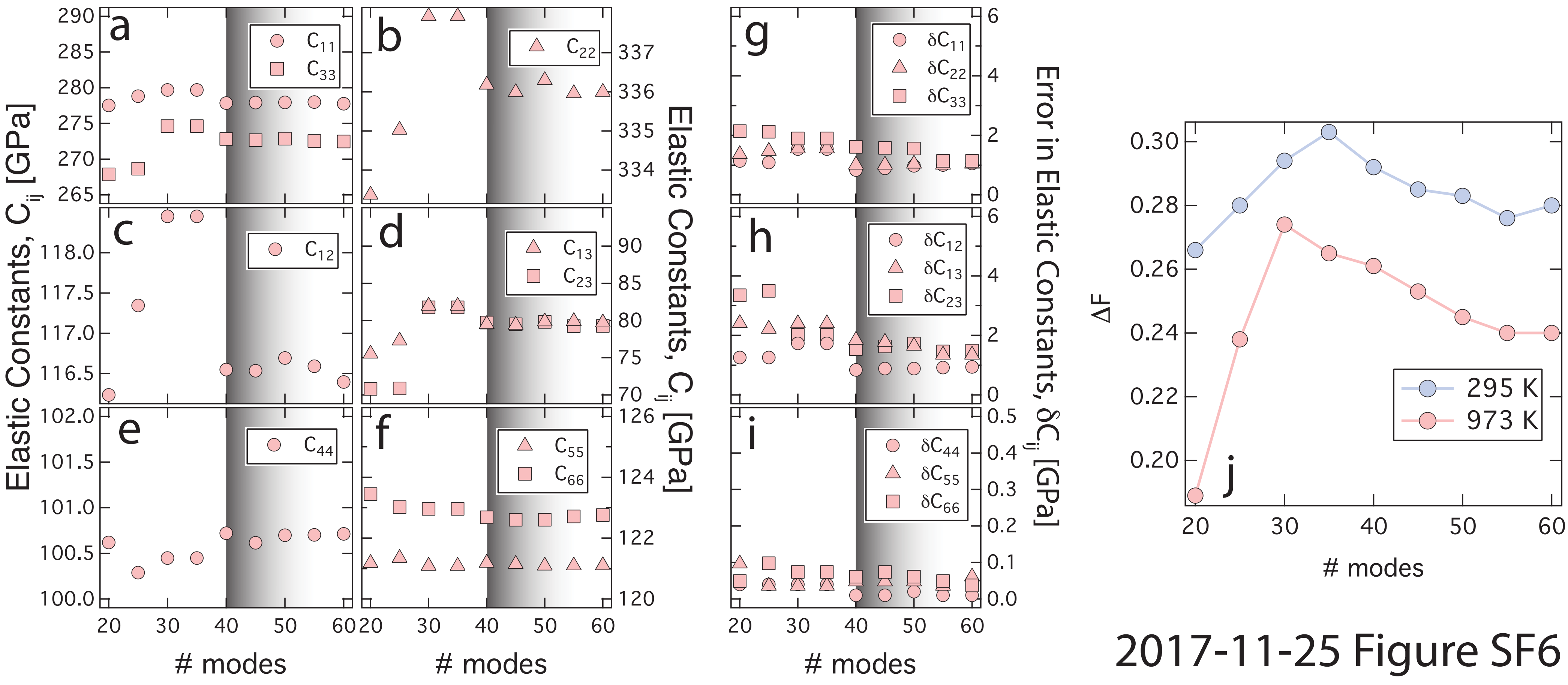

2017-11-25 Figure SF6

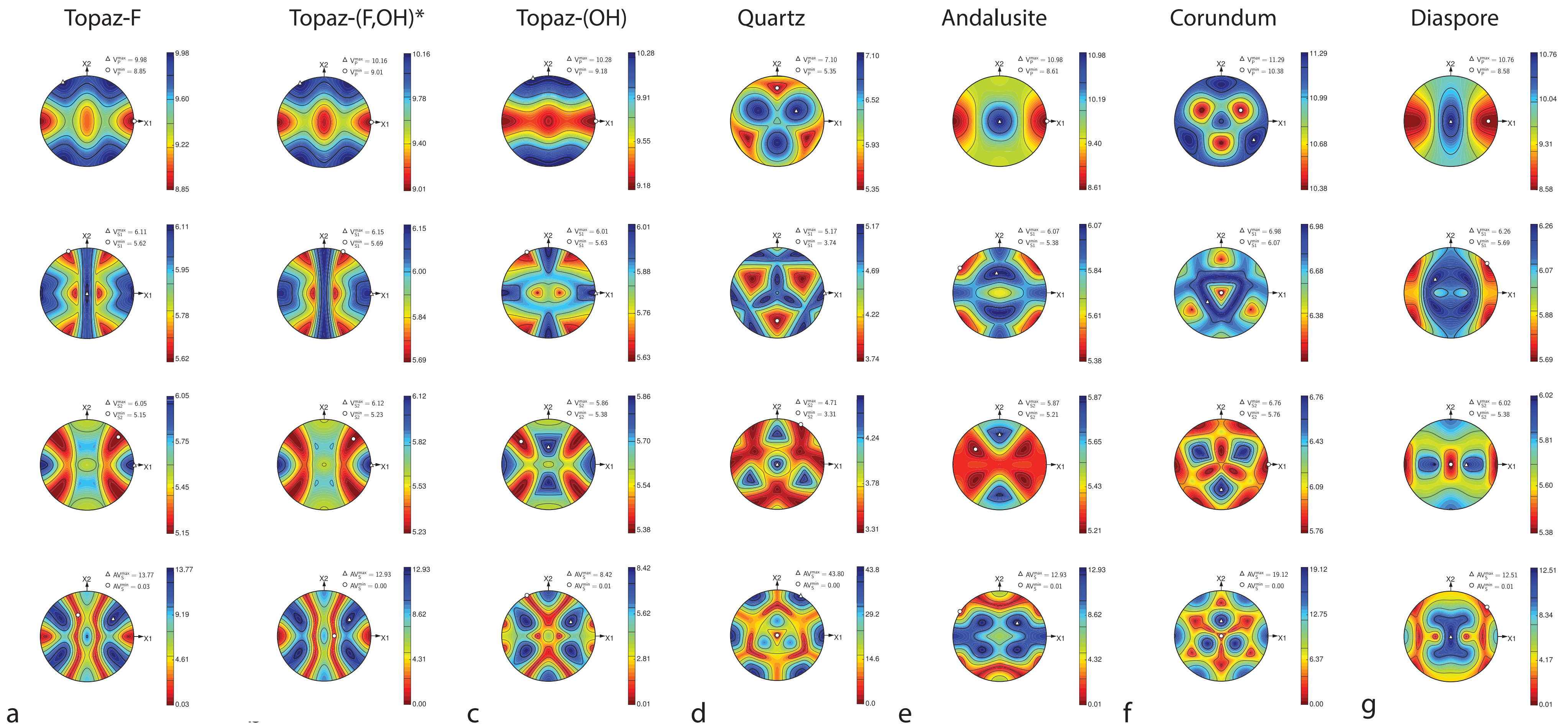

2017-11-25 Figure SF7

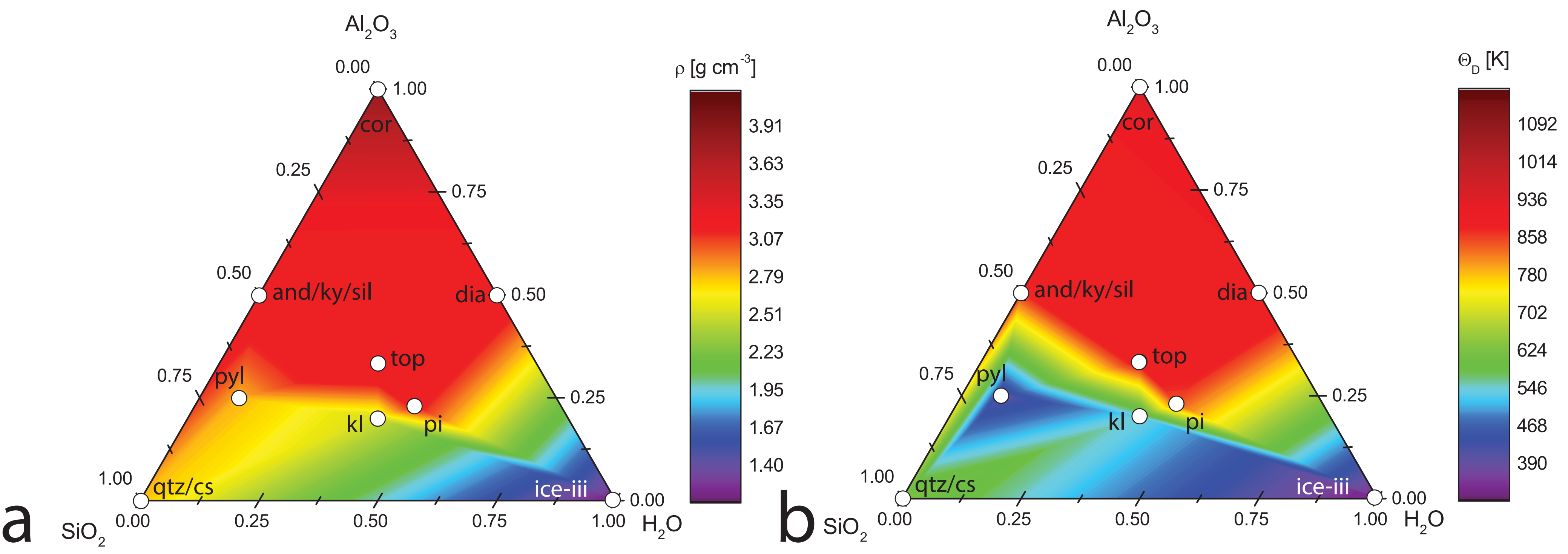

2017-11-25 Figure SF8
